# Blood Flow Restriction during Walking Does Not Impact Body Composition or Performance Measures in Highly Trained Runners

**DOI:** 10.3390/jfmk9020074

**Published:** 2024-04-13

**Authors:** Ashley A. Herda, Christopher J. Cleary, Dana Young, KathleenMae B. Rogers, Santiago E. Umana Segura, Christopher Bernard, Lisa M. Vopat, Bryan G. Vopat

**Affiliations:** 1 Department of Health, Sport, and Exercise Sciences, University of Kansas, Lawrence, KS 66046, USA; cj.cleary@ku.edu (C.J.C.); kathleenmaerogers@gmail.com (K.B.R.); santiu5530@ku.edu (S.E.U.S.); 2 Department of Orthopedics and Sport Medicine, University of Kansas Medical Center, Overland Park, KS 66213, USA

**Keywords:** vascular occlusion, aerobic, treadmill, tourniquet

## Abstract

Blood flow restriction (BFR) is a commonly used training modality that has been demonstrated to enhance muscle characteristics such as size and function. The purpose of this study was to determine if a 4-week walking program with or without BFR in healthy, active adults has an effect on body composition, anaerobic, and aerobic running performance. Thirty-three participants, randomized among three groups, completed the walking program, which included five sets of 2 min walking intervals with 1 min rest, with or without BFR, or 10 min walking with BFR. Assessments completed before and after the walking program included body composition, 40-yard sprints, and a VO_2MAX_ test on a treadmill. A two-way ANOVA revealed no changes among the groups nor for any variables at any time (*p* > 0.05). Additionally, one main effect for time indicated the VO_2_ at V-slope threshold was greater following training for all groups combined (*p* = 0.001). The results demonstrate that low volume and intensity walking with BFR for 4 weeks did not provide a sufficient stimulus for changing body composition or performance metrics in a group of very active adults. Longer or more isolated exposure of BFR on the limbs may contribute to more pronounced adaptations.

## 1. Introduction

Blood flow restriction (BFR) is a training method that originated in the 1960s, alternatively called Kaatsu training, and was normalized in the 1990s [1]. This method uses a cuff that can rapidly inflate to a specified pressure set to partially occlude arterial blood flow and restrict venous return from within working musculature during exercise [1,2,3]. Some methods of BFR include automated cuffs which include internal sensors that determine maximal occlusion pressure and then release pressure to allow partial arterial inflow to a prescribed level. Additionally, other cuff sets include manual detection of occlusion pressure using doppler and manual inflation which may take slightly longer to get to the desired inflation and occlusion pressure. BFR training (BFRT) has become a widely used adjunct to strength training in the athletic population due to its ability to stimulate muscle hypertrophy with relatively lower intensity exercises [4]. BFRT at 20–50% of 1-repetition maximum (RM) has been reported to elicit slightly lower improvements in muscular strength and hypertrophy that can be gained at traditional 70–90% 1-RM loads [5]. These changes elicited by either mode of resistance exercise are greater than no exercise control conditions. In addition, BFRT is used within physical therapy and rehabilitation settings to promote strength and hypertrophy using low loads, specifically when patients cannot fully load their injured limb [6]. The limited blood flow to the limb creates a hypoxic environment within the working muscles and ultimately leads to cell swelling that stimulates protein synthesis through anabolic pathways [7], as well as angiogenesis from rapid reperfusion following cuff release [8]. The full body and segmental body composition changes incurred from BFRT are not always directly evaluated. Rather, only local adaptations are measured using magnetic resonance imaging, computed topography, or ultrasound [9,10].

BFRT research has predominantly focused on the musculoskeletal response, intramuscular adaptations, and anaerobic performance; however, there have been few studies investigating BFRT’s effect on the cardiovascular system, pulmonary system, and overall performance measures in the active population, with some conflicting evidence supporting or refuting its utility [8,11,12,13]. Some of these studies have used BFR during resistance exercise (BFR-RT) [11,14,15,16] and others with BFR during aerobic exercise (BFR-AT) [17,18]. Use of BFRT during low-load-resistance exercise or low intensity cardiovascular exercise could potentially allow athletes an avenue to improve their fitness while recovering from injury. Current research also lends support to cardiovascular adaptations and endocrine responses with the use of BFR due to the restriction of venous return with cuff inflation [7,15,19,20]. Vasculature has also been reported to be affected with the use of BFRT with hyper perfusion and hyperoxygenation following cuff release, ultimately leading to angiogenesis [6,8,21]. Some noted chronic adaptations to the use of BFR during aerobic exercise include a 9% increase in aerobic capacity in older women after 10 weeks [22], among other changes to musculature (activated AKT-mTOR pathway; myogenesis). Many studies are shorter duration (2–6 weeks) and all reflect similar responses [8,9,23]. A thorough review of all applications and effects of various modes of BFR is presented by Freitas et al. [2].

Overuse injuries, such as stress fractures, are common among runners and high-level endurance athletes, as they attempt to continuously improve their performance with increased training and at high intensities [24]. Athletes that suffer from injuries and suddenly reduce their training volume experience a rapid decline in their cardiovascular and pulmonary fitness, and early return to high-impact activity is often contraindicated. Acute evidence suggests oxygen consumption (VO_2_) and heart rate [2,8] is elevated during low-load BFR-AT; however, these studies lack a clear indication if that elevated VO_2_ exceeds the anaerobic threshold to enhance VO_2_ with chronic or prolonged training. The primary reason is that VO_2_ parameters are not recorded during the training sessions.

The existing literature shows significant variability in BFRT training mode (BFR-AT or BFR-RT), duration, intensity, cuff type and size, occlusion pressure (40–100% limb occlusion pressure), and occlusion time in various athletic samples, and due to this, the ability to extrapolate results to recreationally active adults becomes challenging. Therefore, the present study aims to determine if a 4-week walking program with or without BFR in healthy, active adults has an effect on anaerobic and aerobic running performance, as measured by a 40-yard dash, maximal oxygen consumption metrics, and time to exhaustion. We hypothesize that given the systemic effects of blood flow restriction, we will see a change in these performance measures following a low-volume and -intensity walking program. A secondary analysis was to see if any body composition changes occur as a result of the low-intensity walking program.

## 2. Materials and Methods

### 2.1. Study Design

In a randomized, parallel, controlled design, subjects were randomly assigned to 1 of 3 walking groups with or without BFR and the matched active time was 10 min of total walking three days per week for 4 weeks. Before (PRE) and after (POST) the walking intervention participants were assessed for body composition [body mass (BM; kg), lean body mass (LBM; kg), skeletal muscle mass (SMM; kg), leg lean mass (LLM; kg), skeletal muscle index (SMI; kg/m^2^), fat mass (FM; kg), and percent body fat (F_pct_; %)] using bioelectrical impedance analysis (BIA), sprint performance [40-yard fly sprint time (Dash40; s) and speed; m/s], and aerobic capacity [relative and absolute VO_2MAX_ (VO_2REL_; mL/kg/min and VO_2ABS_; L/min), time to exhaustion (T_MAX_; s), V-slope threshold (VT_VO2_; L/min) for submaximal oxygen consumption, and expressed as a percent of max (VT%)]. After a self-selected 5 min warm up on a stationary bike, elliptical, or dynamic stretching, participants completed 2 40-yard fly sprints, followed by a maximal aerobic capacity test on a treadmill using indirect calorimetry. All participants were informed of potential risks of both blood flow restriction and cardiovascular activity and signed a written form of informed consent to participate in the study. Interested participants were screened for the following inclusion and exclusion criteria: all study procedures were approved by the university’s institutional review board for human subject research (#STUDY00145843) and each subject signed an approved informed consent form prior to completing any study-related activities.

### 2.2. Subjects

Forty-four healthy, active males (*n* = 26; mean ± SD; age = 32.9 ± 11.0 yrs; height = 181.0 ± 8.5 cm; body mass = 86.5 ± 19.4 kg) and females (*n* = 18; age = 34.1 ± 10.0 yrs; height = 166.8 ± 6.7 cm; body mass = 60.6 ± 11.3 kg) volunteered for this study. An a priori sample size set to provide 80% or greater power at an α of 0.05 and moderate effect size of 0.25 was identified using G*Power (Version 3.1.9.6, Kiel, Germany) at 10–12 participants per group. To allow for attrition, 15 participants per group were recruited and a parallel block randomization tool pre-identified subject ID numbers into one of 3 groups (1: BFR_INT_; 2: BFR_10_; 3: CON_10_). Participant ID numbers were allocated as the individuals arrived at the sports performance facility and all participants completed the study assessments before (PRE) and after (POST) the 4-week intervention period. Recruited participants met the inclusion criteria of healthy, active (>125 min/week moderate physical activity for the past 6 months) males and females between 18 and 40 years old, inclusive. All participants were screened for pertinent medical history including but not limited to heart disease, pulmonary disease, respiratory dysfunction, cancer, or musculoskeletal injuries that would exclude them from participation in the study. All participants provided their current exercise (recreational or competitive) regimen and were instructed to maintain their current exercise and dietary habits throughout the duration of the study.

### 2.3. Procedures

#### 2.3.1. Body Composition Assessment

Participants were asked to visit the research facility after having fasted for at least 4 h prior to their scheduled time and rested (>24 h from any previous exercise exertion). All subsequent visits for each participant were completed at the same time of day and they were all asked to limit food consumption 4 h prior and arrive rested and hydrated. Body Composition procedures included standing height (cm) without shoes on a platform stadiometer (InBody 170B; Cerritos, CA, USA) and were followed by bioelectrical impedance analysis (BIA; InBody 770, Cerritos, CA, USA) for total body and segmental muscle mass. Participants stood barefoot on the BIA platform after removing all metal and extra layers of clothing and cleaning their hands and feet with a conductive wipe.

#### 2.3.2. Sprint Test

Participants were then instructed to complete a 5 min self-selected warm up using a treadmill, turf field, or dynamic stretching. Participants completed light jogging in or outside, depending on the weather and dynamic stretches in the order of their choosing. This was not standardized so the athletes could warm up as they were accustomed to prior to a race or competitive event. Two sets of timing gates (Brower TCi WirelessTiming System, Draper, UT, USA) were arranged 40 yards apart on a turf field with 5-yard line marks. An additional 10 yards were available before and after the 40-yard course for acceleration and deceleration, respectively. Participants were instructed to utilize the first 10 yards to try and achieve maximal speed and to sprint as fast as possible through the two sets of timing gates. Time started when the participant passed through the first set and stopped as they crossed the second set of gates and average speed in meters per second were derived from the sprint time and distance. Three trials were completed, with the first at 50–75% speed and the next two at maximal speed, all with approximately 3 min of rest time and active recovery (walking back to the start) between each attempt.

#### 2.3.3. Graded Exercise Test and Indirect Calorimetry

Participants were provided a chest-strap heart rate monitor (Polar H10; Polar Electro, Inc., Kempele, Finland) to wear throughout the graded exercise test (GXT). Participants’ resting heart rate was monitored as the treadmill test was explained to them, including the Borg scale rating of perceived exertion (RPE) [25]. Once the headgear and mouthpiece (Hans-Rudolph, Inc., Shawnee, KS, USA) were secured and comfortable on the participant, they were guided to step up onto the treadmill (Woodway 4Front, Woodway USA, Inc., Waukesha, WI, USA) and a tube was connected to the mouthpiece and the TrueOne 2400 metabolic cart (ParvoMedics, Sandy, UT, USA) was used to collect and analyze expired gases during the GXT. The GXT protocol included a 5 min warm-up at 0% incline, where the first 2 min was walking at 3 mph (4.83 km∙h^−1^) followed by 2 min at 5 mph (8.05 km∙h^−1^) and the final warm-up minute allowed the participant to identify a comfortable running pace. The speed established by the participant at the end of the 5 min warm-up was then used for the remainder of the GXT and the incline increased 2% per minute until a plateau in VO_2_ was identified or volitional exhaustion. Test termination criteria for establishing a true VO_2MAX_ were used based on ACSM exercise testing guidelines with at least two of the following criteria: (a) plateau in heart rate (HR) or HR values within 10% of the age-predicted HRmax, (b) RPE above 17 on the 6–20 Borg scale, (c) plateau in VO_2_ (less than 150 mL·min^−1^), and/or (d) RER value greater than 1.1. Heart rate and RPE were documented within the last 15 s of each stage and at test termination as well as throughout a 3 min walking cool-down. Following the manufacturer guidelines, the metabolic system was calibrated daily, within 30 min prior to the test commencing and throughout the day in the event of multiple testing sessions in a single day. Running speed remained constant from PRE- to POST-testing for each participant. Variables analyzed were documented from exported metabolic reports at each visit. Additionally, the anaerobic threshold was documented using the V-slope method and was considered the inflection or breaking point of expired VCO_2_ with increased intensity and VO_2_ utilization [26].

#### 2.3.4. Walking Training and Blood Flow Restriction

Upon completion of the PRE-testing visit, participants were scheduled to complete walking training 3 days per week for 4 weeks, or 12 walking sessions. Using computer-generated randomized group allocation, they were each assigned to one of 3 groups. BFR_INT_ completed walking with BFR at 80% arterial occlusion pressure for 2 min intervals, separated with 1 min rest, five times, following similar protocols to Renzi et al., Loenneke et al., and Abe et al. [8,23,27]. The cuffs were inflated for the duration of the session using a 10 cm wide cuff on the proximal thigh of each leg (Smart Cuffs 3.0, Strongsville, OH, USA). Limb occlusion pressure was established using a handheld vascular Doppler (SD3 Vascular, Edan Instruments, San Diego, CA, USA) to determine when full occlusion occurred (no pulse wave sound) at the posterior tibial artery, just posterior to the medial malleolus. The cuff pressure was then released to 80% of that value or with the automated cuff inflation (version 3.0) and built in sensor. BFR_10_ completed walking with 80% occlusion for 10 min straight. CON_INT_ performed repeated walking intervals without BFR, totaling 10 min of active walking. The speed for all walking sessions was set at 3 mph (4.83 km∙h^−1^). Session RPE via Borg CR-10 [28] was documented for all training visits. All participants were asked to maintain their daily exercise routines throughout the duration of the study.

### 2.4. Statistical Analyses

Mean daily and weekly physical activity estimates were determined from participants’ self-reported values. All variables were compared across the group at PRE using one-way analysis of variance (ANOVA) to determine if any baseline differences existed. Subsequently, separate two-way (group × time) multifactorial ANOVAs were conducted to determine any interaction and main effects for time and group if no interaction existed. Effect sizes were indicated as partial eta squared (*η_p_*^2^) for the ANOVA interactions, interpreted as trivial (<0.01), small (0.01–0.06), moderate (0.06–0.14) or large (>0.14). All calculations were conducted in SPSS v.28 (IBM SPSS Statistics, Armonk, NY, USA) and determined significant at an α of 0.05.

## 3. Results

### 3.1. Participant Characteristics

Participants aged 18–49 years old (30.0 ± 10.09) had engaged, on average, in 393.4 ± 197.2 min per week in physical activity and would be considered very active, with an average daily physical activity level of 56.2 ± 28.2 min daily (range = 21.4–96.4 min), which is in excess of the daily recommended 30 min 5 days per week [29]. Forty-four participants completed all baseline assessments, five participants were omitted due to exclusion criteria and six participants dropped out from the training due to various reasons.

Evaluation of baseline characteristics revealed no significant differences between groups for any of the dependent variables (Table 1).

### 3.2. Body Composition

As indicated in Table 1, there were no significant interactions for body composition variables (*p* = 0.120–0.738; *η_p_*^2^ = 0.020–0.132), no main effects for time (*p* = 0.757–0.980), and no main effects for group (*p* = 0.161–0.711).

### 3.3. Anaerobic Performance

Sprint speed and Dash40 performance indicated no two-way interaction (*p* = 0.512 and 0.513; *η_p_*^2^ = 0.043–0.044, respectively). There were also no main effects indicated for time (*p* = 0.328 and 0.324) or group (*p* = 0.792 and 0.821), for sprint speed and Dash40, respectively.

### 3.4. Aerobic Performance

There were also no significant two-way interactions for aerobic performance metrics (*p* = 0.328–0.870, *η_p_*^2^ = 0.009–0.072). There was one main effect for time for VT_VO2_ (*p* = 0.001), which indicated all groups improved from PRE to POST (mean change ± SE = 0.182 ± 0.05 L/min^−1^). There were no other main effects for time (*p* = 0.060–0.409) or group (*p* = 0.112–0.933).

## 4. Discussion

The present study aimed to identify the effect of a 4-week walking program with or without BFR in active healthy adults on body composition, anaerobic, and aerobic running performance, as measured by a 40-yard dash, maximal oxygen consumption metrics, and time to exhaustion. There was no effect of walking training from any condition of walking and only a slight increase in VT_VO2_ across all groups. The potential limiting factors may be twofold. The initial trained status of the participants was very high, as most of the participants reported being endurance athletes (marathon and triathlon completers), as well as the relatively low-intensity stimuli from the training. Participants in the BFR-AT-involved groups indicated an RPE of up to 6 out of 10, suggesting the training was less challenging to this particular group of individuals.

The existing literature on the effects of BFRT on aerobic capacity has wide-ranging training protocols and results, making its implementation difficult. Following a 4-week walking program with or without BFR-AT, older adults that walked at 40% of maximal oxygen consumption (VO_2MAX_) for 15 min with blood flow occlusion to both legs improved not only quadriceps muscle volume and cross-sectional area but also improved VO_2MAX_ and time to exhaustion [9]; this may have been an intensity greater than experienced by the present participants and their participants were lesser trained. As is a limitation of the present study, neither heart rate nor oxygen consumption were monitored during the training sessions, only session RPE. Additionally, a 3-week walking program in aerobically active young males utilizing BFR-AT (twice daily, 6 days per week) with the Kaatsu method was reported to increase cross sectional area of the quadriceps and hamstrings, muscle volume of the quadriceps, hamstrings and adductors, and improved one-repetition maximum strength for leg press and leg curl [23], where the present results did not indicate any body composition or muscular changes. Differences in the volume of training (36 sessions vs. 12 sessions) may have influenced the reported outcomes. Furthermore, a 2-week walk training program with BFR-AT, male college basketball players demonstrated increased stroke volumes and decreased heart rates post-training, following five 3 min bouts at 4–5 km/h (2.5–3 mph) and 5% incline, lending further support to the positive cardiovascular adaptations that may occur with BFRT [30], However, these are metrics not assessed in the present study. These basketball players also demonstrated significant improvements in VO_2MAX_, maximal minute ventilation, and anaerobic capacity/mean power when compared to their counterparts that did not use BFR-AT. Again, the greater intensity using an incline of 5% during walking at a similar speed as the present study may have contributed to their noted results.

The isolation of load and duration of BFR exposure on the working musculature may be a driving factor in adaptations reported by others [19,23]. Using different exercise stimuli, a 5-week rowing program with BFR resulted in highly trained participants improving their VO_2MAX_, but did not have any notable improvements in their squat one repetition maximum [31]. Paton et al. [17] reported the effects of BFR-AT on aerobic capacity, found an improvement in peak running velocity, submaximal oxygen cost, and time to exhaustion during running in those that performed running training with BFR. However, similar to the present study, the authors found no significant differences between maximal oxygen consumption between their test and control groups. Furthermore, authors attribute improvement in these parameters not to aerobic adaptations but more to improvement in muscle strength and the ability of the muscles to adapt to the increased stress placed upon them due to occlusion of blood flow during training, with the training stimulus being running at a high intensity, not walking [17].

Overall, the training status of the present study participants was higher than those reported in other studies, which may have influenced the limited change in the present results. Additionally, not all physiological explanations of change, or lack of, were assessed as they were beyond the scope of this study.

Apparent limitations also exist in the present study, including the block randomization strategy did not account for equal distribution of females across groups. Although this may have impacted group means, the highly trained status of the participants would be equivocal among groups considering some of the relative variables, such as, VO_2REL_ and VT_VO2_. Additionally, session VO_2_, heart rate, lactate, and blood pressure were not evaluated therefore any conclusion as to the intensity or systemic load of walking with BFR could not be evaluated beyond the participant-reported session RPE. The walking speed of the present study was comparable to other studies [8,23,27], yet the varying volume (2–3× greater quantity of sessions), intensity (walking uphill), and training status (untrained or lower relative aerobic fitness) compared to those studies would have influenced the outcomes. Using individualized intensities relative to the participant’s VO_2MAX_ may contribute to enhanced adaptation.

## 5. Conclusions

The results demonstrate that walking with BFR for 4-weeks did not provide sufficient stimulus for changing body composition or performance metrics in a group of very active adults, primarily due to the low volume and intensity of the walking activity. Longer or more isolated exposure of BFR on the limbs may contribute to more pronounced adaptations. Consistent evaluation of the same variables, performance metrics and standardized BFRT protocols per mode of exercise (cycling, walking, rowing, running, etc.) should be established to provide comparable results in the future.

## Figures and Tables

**Table 1:** Participant characteristics, body composition, and performance outcomes.

				BFR_INT_		BFR_10_		CON_10_	
				*n* = 11; F = 6		*n* = 11; F = 5		*n* = 11; F = 2	
				Mean	SD	Mean	SD	Mean	SD
General Descriptives	Age (yrs)		30.73		11.17	33.55	10.27	33.82	11.17
	Height (cm)		174.57		13.12	169.76	8.60	178.57	8.98
	Total Body Mass (kg)	PRE	71.04		14.89	69.61	14.94	80.46	13.72
		POST	71.09		13.96	69.17	15.19	80.90	14.39
	BMI (kg/m^2^)	PRE	23.04		2.24	23.84	2.86	25.17	3.20
		POST	23.07		1.99	23.76	2.94	25.29	3.35
Body Composition	Lean Body Mass (kg)	PRE	57.65		12.26	56.18	14.70	62.83	11.57
		POST	57.44		11.66	55.62	14.19	63.66	12.64
	Skeletal Muscle Mass (kg)	PRE	32.43		7.45	31.58	8.79	35.62	7.13
		POST	32.34		7.16	31.34	8.60	36.08	7.77
	Body Fat (%)	PRE	18.66		7.80	20.62	7.01	21.76	8.10
		POST	19.09		7.23	20.42	7.62	21.31	7.85
	Fat Mass (kg)	PRE	13.39		6.86	13.43	4.59	17.63	7.09
		POST	13.64		6.37	13.56	5.30	17.23	6.53
	Leg Lean Mass (kg)	PRE	18.24		4.44	16.71	3.90	19.34	3.36
		POST	18.17		4.20	16.59	3.83	19.56	3.39
	Skeletal Muscle Index (kg/m^2^)	PRE	7.87		0.93	7.75	1.23	8.20	0.92
		POST	7.84		0.83	7.72	1.19	8.28	1.01
Anaerobic Performance	Sprint Speed (m/s)	PRE	5.77		0.72	5.62	0.59	5.55	0.86
		POST	5.71		0.79	5.55	0.51	5.56	0.90
	40-yard Dash Time (s)	PRE	14.38		1.83	14.71	1.61	15.07	2.26
		POST	14.59		2.06	14.86	1.42	15.01	2.16
Aerobic Performance	Absolute VO_2_ (L/min)	PRE	3.32		0.70	3.42	1.22	3.78	0.76
		POST	3.33		0.71	3.49	1.21	3.87	0.69
	Relative VO_2_ (mL/kg/min)	PRE	47.05		6.75	48.13	9.22	47.16	8.16
		POST	47.17		7.32	49.42	10.05	48.19	7.13
	Time to Exhaustion (s)	PRE	310.82		78.09	311.82	67.33	310.36	89.85
		POST	310.18		78.27	327.91	69.14	334.00	93.59
	V-Slope Threshold (%)	PRE	70.91		9.53	65.91	10.06	72.18	3.03
		POST	74.55		10.75	65.91	14.92	73.18	7.81
	VO_2_ at VT (L/min)	PRE	2.37		0.56	2.33	0.94	2.76	0.50
		POST	2.57 *		0.66	2.53 *	0.93	2.91 *	0.46
VO_2MAX_ Test Terminal Effort	Speed (mph)	Pre & Post	6.53		1.30	6.67	0.81	6.61	0.84
	Incline (%)	PRE	10.72		2.72	10.82	2.40	10.54	3.24
		POST	11.27 *		2.72	12.27 *	2.53	11.27 *	3.13
	Heart Rate (bpm)	PRE	187.36		8.16	180.45	10.88	184.36	10.62
		POST	186.09		7.83	182.73	9.96	185.27	9.06
	Rating of Perceived Exertion	PRE	19.0		1.10	18.81	1.17	19.55	0.69
		POST	19.0		1.18	19.64	0.67	19.0	1.18
	Respiratory Exchange Ratio	PRE	1.11		0.07	1.09	0.07	1.11	0.05
		POST	1.13 *		0.08	1.13 *	0.06	1.13 *	0.04
	Training Session RPE	(Range)	5.0 ± 1.0		(4–6)	4.0 ± 2.3	(4–6)	2.0 ± 1.2	(1–4)

All data are presented as mean and standard deviation in italics. * denotes a significant difference from PRE collapsed across groups.

## Data Availability

The raw data supporting the conclusions of this article will be made available by the authors on request.
